# Big Data Analytics for Genomic Medicine

**DOI:** 10.3390/ijms18020412

**Published:** 2017-02-15

**Authors:** Karen Y. He, Dongliang Ge, Max M. He

**Affiliations:** 1 Department of Epidemiology and Biostatistics, Case Western Reserve University, Cleveland, OH 44106, USA; kyh9@case.edu; 2 BioSciKin Co., Ltd., Nanjing 210042, China; 3 Computation and Informatics in Biology and Medicine, University of Wisconsin-Madison, Madison, WI 53706, USA

**Keywords:** Big Data analytics, clinically actionable genetic variants, electronic health records, healthcare, next-generation sequencing

## Abstract

Genomic medicine attempts to build individualized strategies for diagnostic or therapeutic decision-making by utilizing patients’ genomic information. Big Data analytics uncovers hidden patterns, unknown correlations, and other insights through examining large-scale various data sets. While integration and manipulation of diverse genomic data and comprehensive electronic health records (EHRs) on a Big Data infrastructure exhibit challenges, they also provide a feasible opportunity to develop an efficient and effective approach to identify clinically actionable genetic variants for individualized diagnosis and therapy. In this paper, we review the challenges of manipulating large-scale next-generation sequencing (NGS) data and diverse clinical data derived from the EHRs for genomic medicine. We introduce possible solutions for different challenges in manipulating, managing, and analyzing genomic and clinical data to implement genomic medicine. Additionally, we also present a practical Big Data toolset for identifying clinically actionable genetic variants using high-throughput NGS data and EHRs.

## 1. Introduction

Next-generation sequencing (NGS) technologies, such as whole-genome sequencing (WGS), whole-exome sequencing (WES), and/or targeted sequencing, are progressively more applied to biomedical study and medical practice to identify disease- and/or drug-associated genetic variants to advance precision medicine [1,2]. Precision medicine allows scientists and clinicians to predict more accurately which therapeutic and preventive approaches to a specific illness can work effectively in subgroups of patients based on their genetic make-up, lifestyle, and environmental factors [3]. To date, over 6000 Mendelian disorders have been studied at the genetic level [4,5] and over 1500 clinically-relevant complex traits have been studied with genome-wide association study (GWAS) approaches [6]. Clinical research leveraging electronic health records (EHRs) has become feasible as EHRs have been widely implemented [7]. Additionally, a number of studies have been designed to combine genomic and EHR data to improve clinical research and/or healthcare outcome (Table 1).

Leveraging large-scale genomic data with comprehensive clinical data derived from EHRs can implicate disease- and/or drug-associated variants for individualized diagnosis and therapy. NGS technological advancements in clinical genome sequencing and the adoption of EHRs will very likely create patient-centered precision medicine in clinical practice. Genomic data generated by NGS technologies are a vital component in supporting genomic medicine, but the volume and complexity of the data raise challenges for its use in clinical practice [8]. For instance, sequencing a single whole genome generates more than 100 gigabytes of data. Therefore, the development of novel bioinformatics infrastructures is required to implement NGS in clinical practice.

Big Data is a term used to describe data sets with such large volume or complexity that conventional data processing methods are not good enough to deal with them. Big Data has been described disparately by different people [9]. The most popular definition of Big Data is the 5Vs, which are Volume, Velocity, Variety, Verification/Veracity, and Value [10]. The definition of Big Data might be subjected to technological advances in the future. Big Data infrastructure is a framework, which covers important components including Hadoop (hadoop.apache.org), NoSQL databases, massively parallel processing (MPP), and others, that is used for storing, processing, and analyzing Big Data. Big Data analytics covers collection, manipulation, and analyses of massive, diverse data sets that contain a variety of data types including genomic data and EHRs to reveal hidden patterns, cryptic correlations, and other intuitions on a Big Data infrastructure [11]. Due to its effectiveness, Big Data analytics is widely used in different research fields [12]. In this review, we describe how one type of Big Data, genomic data, is applied to improve clinical research and healthcare. We give an overview of the challenges in processing genomic data and EHRs, provide possible solutions to overcome these challenges using approaches that ensure the safety of genomic data, and present a Big Data solution for identifying clinically actionable variants in sequence data. We also discuss the requirement for the efficient integration of genomic information into EHRs.

## 2. Challenges of Handling Genomic and Clinical Data

### 2.1. Challenges in Manipulating Genomic Data

**Figure 1:**
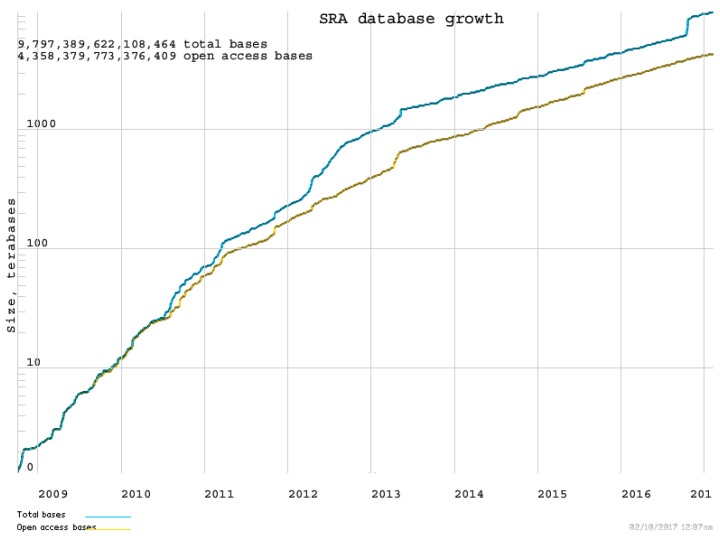
The SRA database growth in the past eight years.

**Figure 2:**
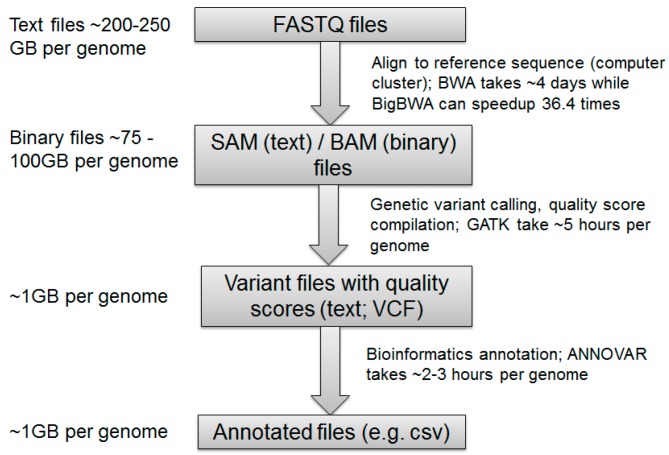
The approximately files sizes of different NGS data formats and running times of generating those different format files. BWA: Burrows-Wheeler aligner, GATAK: genome analysis toolkit, BAM: the binary version of sequence alignment/map, FASTQ: a text-based format for representing either nucleotide sequences or peptide sequences, VCF: variant call format.

Although more than 6000 Mendelian disorders have been studied at the genetic level so far, we still do not have a clear understanding of the majority of their roles in health and diseases [25]. Over the past eight years, the size of the NIH sequence read archive (SRA) database has grown exponentially (Figure 1). While the development of NGS technologies has made it increasingly easier to sequence a whole genome or exome, there continue to be considerable challenges in terms of handling, analyzing, and interpreting the genomic information generated by NGS. Since there are over three billion base pairs (sites) on a human genome, sequencing a whole genome generates more than 100 gigabytes of data in BAM (the binary version of sequence alignment/map) and VCF (Variant Call Format) file formats. The actual size of a BAM file is determined by the coverage (the average number of times each base is read; read depth) and read length in a sequencing experiment. Given a 30× WGS data for a single sample, the size of its FASTQ file can be approximately 250 GB, the BAM file can be approximately 100 GB, the VCF file can be about 1 GB, and the annotated files can be approximately 1 GB as well. The approximate file sizes of different NGS data formats and running times of generating those different format files are described in Figure 2. Big Data infrastructures can greatly facilitate the analysis of these data. For example, Big Data-based Burrows-Wheeler Aligner (BWA) can increase the alignment speed 36-fold compared to the original BWA [26]. Currently, most analytical methods for sequencing data use VCF files that assume all “no-call sites” are the same as reference alleles. In fact, many “no-call sites” may be caused by low quality coverage. Therefore, the data quality information, such as coverage and Phred-scaled base quality scores for every site, needs to be utilized to pinpoint whether “no-call sites” are reference-consistent with high coverage or reference-inconsistent caused by low coverage in the downstream data analysis [27]. A number of toolsets for data compression, cloud computing, variant prioritization, copy number variation (CNV) detection, data sharing, and phenotypes on exome sequencing data have been reviewed by Lelieveld et al. [28]. Because VCFs are much smaller than BAM files, analytical tools on VCFs may not always require a Big Data infrastructure. However, researchers are currently facing substantial challenges in storing, managing, manipulating, analyzing, and interpreting WGS data for moderate numbers of individuals if they need to take into account of data quality information stored in BAM files. These challenges will become exacerbated when millions of individuals are sequenced, which embodies the goals of the precision medicine initiative (PMI) in the U.S. and similar efforts of the same scale elsewhere in the world. Leveraging the distribution and scalability inherent in Big Data’s infrastructures, it can be feasible to develop a Big Data system to manage and analyze the extensive genomic data compatible with clinical workflows.

### 2.2. Challenges in Manipulating Clinical Data

Up until the previous decade, approximately 90% of clinicians in the U.S. routinely recorded patient medical records by hand and stored them in color-coded files. In the past five years, the percentage of clinicians using certified EHR systems has grown dramatically [29]. Clinical data extracted from the EHRs for each patient can include the international classification of diseases (ICD) codes, drugs, treatments, procedure (CPT) codes, laboratory values, clinician notes, as well as self-reported dietary and physical activity data. The ICD code is a clinical cataloging system utilized by clinics and hospitals to classify and code diagnosis, symptom, procedure, and treatment in the U.S. Not only are ICD codes used for disease classification, but also as medical billing codes [30]. The volume of clinical data extracted from EHRs can be considerable. For example, the EHR data of ~20,000 patients enrolled in the Personalized Medicine Research Project (PMRP) at Marshfield Clinic is approximately 3.3 GB. The elements in clinical data can be used to classify and measure associations between environmental exposures and clinical consequences. An important application of mining clinical data is patient classification [31,32,33,34]. Without stringent and appropriate phenotyping approaches, the classification cannot be appropriately measured, resulting in false positive or negative associations [31]. Machine learning (ML) involves training an algorithm to systematically classify patients into phenotypic groups [35]. To do this, the ML classifier needs to learn which elements in clinical data are providing useful insights for distinguishing the different phenotypic groups. With the proliferation of EHR adoption, computational phenotyping characterization has shown its advantage in classifying research subjects [36]. In addition, millions of data points regarding tens of thousands of clinical elements within the EHRs are available for EHR-based phenotyping. Like sequence data, it will also become a significant challenge to store, manage, manipulate, and mine the complete clinical data of millions of individuals. Therefore, it is necessary to develop advanced and efficient ML approaches for subject characterization and/or better phenotyping. Meanwhile, some ML tasks may take one or two days or even several days to run specific data mining algorithms. For example, to mine large-scale literature, ML approaches on a Big Data infrastructure could be performed 100 times faster than any of the existing ML tools without using any Big Data infrastructures [37].

## 3. Big Data on the Cloud

### 3.1. Cloud Computing

Cloud computing providers offer services that provide the infrastructure, software, and programming platforms to clients, and are accountable for the cost for development and maintenance [38]. Compared to creating and maintaining an in-house database, cloud computing is an economical approach to genomic data management because clients pay only for the services that they need. An example of an open-source framework used to develop infrastructure for processing genomic data in a cloud computing environment is Hadoop. It breaks the data into small fragments, distributes them across many data nodes, delivers the computational code to the nodes so that they are processed in parallel, and collectively assembles the results at the end. The parallel processing of many small pieces of data, known as MapReduce, greatly shortens the computing time. Challenges of using cloud computing for genomic data include lengthy data transfers for uploading NGS data to the cloud server, the perceived lack of information safety in cloud computing, and the requirement for developers with advanced programming skills to develop programs on the Hadoop [38].

### 3.2. Privacy and Security Challenges of Cloud Computing

Cloud computing infrastructures could be deployed with miscellaneous platforms and configurations in which each platform could be configured with diverse security, confidentiality, and authentication settings [39]. These unique aspects could exacerbate security and privacy challenges [40]. Some cloud service providers including Microsoft Azure [41] and Amazon Web Services [42] provide the health insurance portability and accountability act (HIPAA) amenable services for analyzing biomedical data. In addition, data security and privacy in cloud computing is an active research field involving the use of virtual machines [43] and sandboxing techniques [44] for biomedical data management on the cloud.

## 4. Big Data Analytics in Genomic Studies

### 4.1. NGS Read Alignment

NGS involves breaking DNA into large amounts of segments. Each segment is called a ‘read’. Due to biases in sample processing, library preparation, sequencing-platform chemistry, and bioinformatics methods for genomic alignment and assembly of the reads, the distribution and/or length of reads across the genome can be uneven [45,46]. Therefore, some genomic regions are covered with more reads and others with fewer reads. As mentioned previously, read depth denotes the average number of times each base is read. For instance, a 10× read depth means that each base is present in an average of 10 reads. For RNAseq, read depth is more often designated as number of millions of reads. Read alignment involves lining up the sequence reads to a reference sequence [47,48] to allow comparison of sequence data from a sample sequenced with the reference genome. A number of alignment tools including CloudBurst [49], Crossbow [50], and SEAL [51] have been developed on Big Data infrastructures. More programs designed for short-read sequence alignment are shown in Table S1. Alignment allows a number of quality control (QC) measures, such as the proportion of all reads aligned to a reference sequence, the ratio of unique reads aligned to a reference sequence, and the number of reads aligned at a specific locus. These QC measures affect the accuracy of variant calling.

### 4.2. Calling Variants

Variant calling is more reliable with higher read depth, which is especially valuable for detecting rare genetic variants with higher confidence. The read depth needed for accurately calling variants relies on various factors, including presence of repetitive genomic regions, error rate of the sequencing platform, and algorithm used for assembling reads into a genomic sequence. Read depth, such as 100× for heterozygous single nucleotide variant (SNV) detection by WES [52], 35× for genotype detection by WGS [53], and 60× for detecting insertions/deletions (INDELs) by WGS [54], may be required. Some widely-used programs for germline variant calling include SAMtools [55], GATK [56], FreeBayes [57], and Atlas2 [58]. SAMtools comprises a number of utilities for manipulating aligned sequence reads and calling SNV and/or INDEL variants. GATK is a NGS analysis suite designed to identify SNVs and INDELs in germline DNA and RNAseq data. It estimates the likelihood of genotype based on the observed sequence reads at a locus by leveraging a Bayesian model. In addition, it employs a MapReduce infrastructure to accelerate the procedure of processing large amounts of sequence aligned reads in parallel [59,60]. Now, it has been expanded to include somatic variant calling tools by incorporating MuTect [61], and to tackle CNVs and structural variations (SVs) as well. The major difference between SAMtools and GATK is the estimation of the genotype likelihood of SNVs and INDELs for calling variants. Regarding the filtering steps, SAMtools uses predefined filters while GATK learns the filters from the data. FreeBayes is a haplotype-based tool that concurrently discovers SNVs, INDELs, multiallelic sites, polyploidy, and CNVs in a sample, pooled multiple samples, or mixed populations [62]. Atlas2 [58] can be used to analyze data generated by the SOLiDTM platform via logistic regression models trained on validated WES data to detect SNVs and INDELs. This tool can also analyze data generated by the Illumina platform using logistic regression models to call INDELs and a mixture of logistic regression and a Bayesian model to call SNVs [63]. To evaluate various programs/tools, Hwang et al. have systematically examined 13 variant calling programs using gold standard personal exome variants [64].

### 4.3. Variant Annotation

Large amounts of sequence data are being generated by NGS. To pinpoint a small subset of functional variants, many annotation programs have been developed. As one of the most widely used annotation programs, ANNOVAR [65] annotates SNVs, INDELs, and CNVs by exploring their functional consequences on genes, conjecturing cytogenetic bands, and reporting biological functions and various functional scores, including PolyPhen-2 score [66], Sorting Intolerant From Tolerant (SIFT) score [67], the Combined Annotation Dependent Depletion (CADD) score [68], and others. It also discovers variants in conserved regions and identifies variants present in dbSNP [69], the 1000 Genomes Project [70], the NHLBI EPS6500 project [71], and the ExAC [72]. Furthermore, ANNOVAR can employ annotation databases from the UCSC Genome Browser or any other data resources conforming to Generic Feature Format version 3 (GFF3). Other commonly used annotation programs include snpEff [73], and the Ensembl Variant Effect Predictor (VEP) [74]. Xin et al. have developed a web-based service that can be run on the cloud [75]. In order to annotate a WGS data in a short period of time, we are currently developing a cloud-based version of ANNOVAR, which is built on a Hadoop framework and a Cassandra NoSQL database. Additional variant annotation programs are shown in Table S2. In addition, variant annotation depends on biological knowledge in order to provide information on the known or likely impact of variants on gene regulation and protein function [65,73]. To produce a patient report, annotated variants are interpreted in a disease-specific context and are often classified based on their known or expected clinical impact. For instance, the ClinVar [76] variant database, released on 5 July 2016 by the National Center for Biotechnology Information (NCBI), contains 126,315 unique genetic variants with clinical interpretations.

### 4.4. Statistical Analysis of Genomic Data

**Family-based analysis**: Family-based NGS data enable the discovery of disease-contributing *de novo* mutations [77,78,79]. Meanwhile, family-based research strategies can uncover many mutations that may be contributing to recessive, inherited as homozygous or compound heterozygous diseases. SeqHBase [27] is a reliable and scalable computational program that manipulates genome-wide variants, functional annotations and every-site coverage, and analyzes WGS/WES data to identify disease-contributing genes effectively. It is a Big Data-based toolset designed to analyze large-scale family-based sequencing data to quickly discover *de novo*, inherited homozygous, and/or compound heterozygous mutations.

**Population-based analysis**: A number of large-scale population-based sequencing studies are undergoing. For example, the PMI cohort program attempts to sequence one million or more American participants for improving our ability to preclude and cure diseases based on one’s differences in genetic make-up, lifestyle, and environmental factors. By 2025, over 100 million human genomes could be sequenced [80]. Therefore, it is critical to develop statistical toolsets on a Big Data infrastructure for analyzing the genomic data of millions of people.

### 4.5. Security of Genomic Data

Genomic data need to be protected. Therefore, its privacy and confidentiality should be preserved similarly to other protected health information. Privacy safeguards include the utilization of data encryption, password protection, secure data transmission, auditions of data transferring methods, and the operation of institutional strategies against data breeches and mischievous abuse of the data [81]. The Fair Information Practices Principles (FIPPs) offer a framework for enabling data sharing and usage based on the guidelines adopted by the U.S. Department of Health and Human Services [82]. These principles include: individual access, data correction, data transparency, individual choice, data collection and disclosure limitation, data quality and integrity, safeguards, and accountability. The Workgroup for Electronic Data Interchange (WEDI) has released a report outlining the challenges in regards to the infrastructure, workflows, and coordination of health IT integration [83]. These challenges include data access and integration, data exchange, and data governance. Cloud-computing technology advancements offer easier solutions to store large genomic data files and to consolidate data to make them more easily accessible. The use of cloud computing presents extra security concerns because data storage and/or processing services are provided by an entity external to the healthcare organization. Cloud services qualify as a business associate and they must sign a business associate agreement (BAA) in order to adhere to the modifications to the HIPAA privacy, security, enforcement, and breach notification rules [84]. Cloud service providers can address these concerns by including controlled access to the data and building a user role based access system. Additional security measures should be taken, such as protecting the security of the computer network using warning alarms to monitor when changes are made to stored data, and guaranteeing the complete removal of data from its servers if the cloud storage service is no longer being used [39].

## 5. Analysis of Genomic and Clinical Data

### 5.1. Clinically Actionable Genetic Variants

**Figure 3:**
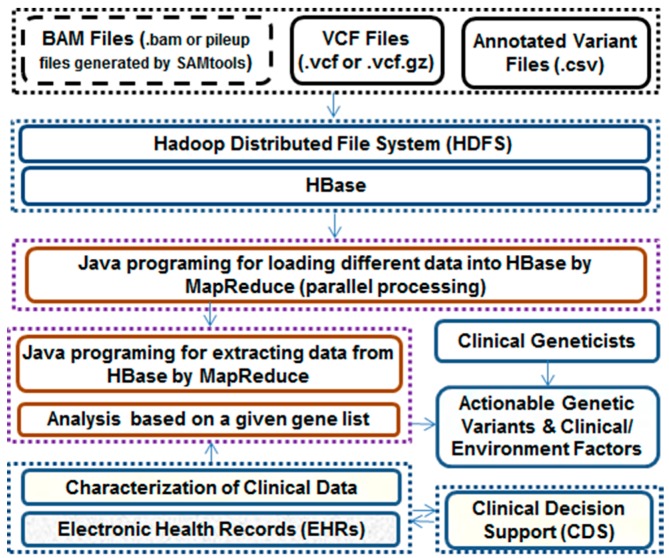
The basic framework of SeqHBase for identifying clinically actionable genetic variants.

In clinical practice, the identification and return of incidental findings (IFs) for clinically disease-contributing variants in a set of 56 "highly medically actionable" genes associated with 24 inherited conditions have been recommended by the American College of Medical Genetics and Genomics (ACMG) [85,86]. A web-based tool for detecting clinically actionable variants in the 56 ACMG genes is developed by Daneshjou et al. [87], and a variant characterization framework for targeted analysis of relevant reads from high-throughput sequencing data is developed by Zhou et al. [88]. SeqHBase [27] is a bioinformatics toolset for analyzing family-based WGS/WES data on a Big Data infrastructure. To deduce biological perceptions from large amounts of NGS data and inclusive clinical data, we have expanded analysis functions within SeqHBase (Figure 3) to detect disease- and/or drug-associated genetic variants quickly.

Even though many variant prioritization tools are available, it remains a challenge to detect clinically actionable variants. Additional efforts are required to distinguish truly clinically actionable variants that can be used to guide clinical decisions. As one variant can be classified as different pathogenicity by multiple clinical laboratories [89], more stringent criteria [90] and the latest ACMG guidelines [91] should be complied to report pathogenic variants [92]. To classify the pathogenicity of new variants, which are not recorded in the ClinVar database [76], and to reach some level of concordance on the clinical variant interpretations, assessments from experts, such as medical geneticists, and/or further biological functional studies are needed. To apply actionable results in clinical practice, genetic findings need to be further complemented with highly strong pathological evidence, along with being reviewed by clinical geneticists.

### 5.2. Clinically Actionable Pharmacogenetic Variants

Substantial efforts have been made to identify clinically actionable pharmacogenetics variants, and it is instructive to review the approach being used. The Coriell Personalized Medicine Collaborative [93], the Clinical Pharmacogenetics Implementation Consortium [94], the Pharmacogenetics Working Group established by the Royal Dutch Association for the Advancement of Pharmacy [95], and the Evaluation of Genomic Applications in Practice and Prevention initiative sponsored by the Centers for Disease Control and Prevention [96] have individually developed similar processes for selecting candidate drugs, reviewing published literature to identify drug-gene associations, scoring evidence supporting associations between genetic variants and drug response, and interpreting the evidence to provide therapeutic guidelines. The approach involving review and interpretation of scientific literature by an expert committee can be considered as the gold standard for determining whether a variant is clinically relevant or actionable, but it also can be costly and labor-intensive. It will not be feasible for experts, either individually or in committees, to review a large number of genetic variants identified in NGS data. Tools such as POLYPHEN-2 [66], VEP [97], Mutation Assessor [98], and SIFT [99] can be used to predict variant effects. However, because these tools are sometimes inaccurate [100] and often differ in their predictions for a same variant [101,102], there will likely be many variants with no clear predicted, clinical interpretation. New methods and toolsets need to be developed to accurately predict the pathogenicity of genetic variants generated by NGS. More importantly, the methods should comply with the U.S. Food and Drug Administration (FDA) guideline [103] and the Clinical Pharmacogenetics Implementation Consortium (CPIC) guideline [104].

## 6. Big Data Analytics in Health Research

Clinical data derived from EHRs have expanded from digital formats of individual patient medical records into high-dimensional data of enormous complexity. Big Data refers to novel technological tools delivering scalable capabilities for managing and processing immense and diverse data sets. On a single level, approaches such as natural language processing (NLP) allow incorporation and exploration of textual data with structured sources. On a population level, Big Data provides the possibility to conduct large-scale exploration of clinical consequences to uncover hidden patterns and correlations [105]. The large amounts of EHRs currently available have enabled us to overcome previously challenging obstacles, such as analyses of rare conditions, more sophisticated analyses, and in-depth analyses of specific data elements [106]. Big Data analytics facilitates the improvement of healthcare from depiction and record to prediction and optimized decision-making.

### 6.1. Health Informatics

In clinical practice, disease characterization is routinely collected from a number of different streams, such as imaging, pathology, genomics, and electrophysiology. However, much of the deeper insights into disease processes and mechanisms remain to be uncovered and interpreted from routinely acquired clinical data. Clinical data of millions of patients at a clinic/hospital or in a large study (e.g., PMI) exhibit many of the features of Big Data. The volume comes from large amounts of records that can be derived from the EHRs for patients; for example, medical images including magnetic resonance imaging (MRI) or neuroimaging data for each patient can be large, while social media data gathered from a population can be large-scale as well. The velocity occurs when data is accumulated at high speeds, which can be seen when monitoring a patient’s real-time conditions through medical sensors for sleep apnea (http://www.sleepapnea.org/), for instance. The variety refers to data sets with a large amount of varying types of independent attributes, such as data sets that are gathered from many different resources. Veracity is a concern when working with possibly noisy, incomplete, or erroneous data where such data need to be properly evaluated using other relevant true evidence. Value portrays the usefulness for improving healthcare outcome. The advance in the fields of health informatics is a vital driving force in the expansion of Big Data, due to either the volume of clinical information produced or the complexity and variety of biomedical data that encompasses discoveries from basic science, translational research, medical system, and population-based study on the determining factors of healthiness. It is essential to develop novel data analytics tools with scalable, accessible and sustainable data infrastructure to effectively manage large, multiscale, and heterogeneous data sets and convert these data into knowledge that can be used for cost-effective decision support, disease management, and healthcare delivery. It is also necessary to develop Big Data infrastructures/systems to store, manage, manipulate, and analyze large-scale clinical data.

### 6.2. Medical Imaging Analysis

Medical imaging data is a type of Big Data in medical research. Imaging genomics is a rapidly growing field derived from recent advancement in multimodal imaging data and high-throughput omics data. The remarkable complexity of these datasets present critical computational challenges. Kitchen et al. reviewed methods for overcoming the challenges associated with integrating genomic, transcriptomic, and neuroproteomic data [107]. There is an increasing interest in integrating neuroimaging data into frameworks for promoting data mining and meta-analyses [108]. In the past century, a central interest in cognitive neuroscience has been trying to understand the human brain [109,110]. Recently, a group of neuroscience researchers used ML methods to map the human brain in order to understand the incredibly complex human cerebral cortex [111]. Human brain mapping is another monumental step toward precision medicine. For example, an important advance in developing operational management and therapeutics of neurological and psychiatric disease empowers researchers to collect and explore data from approximately 100 billion neurons from the brain in a much greater capacity and at an even more rapid speed. As the human brain controls multiple spatial and chronological scales, the data can be used to understand how the brain works by combining all relevant information [112]. Therefore, developing high-performance computing tools based on a Big Data framework becomes critical to neuroscience for improving healthcare [113,114].

### 6.3. Data Sharing

In order to share EHRs across multiple healthcare providers, several key components need to be taken into account: (1) *functional interoperability*, which allows data (e.g., medical records) to be exchangeable from one EHR system to other EHR systems without any restrictions; (2) *structural interoperability*, which permits the data structure to be exchangeable across all systems; (3) *semantic interoperability*, which allows multiple systems to exchange data and to easily make use of the data exchanged; and (4) *interpretation*, which allows clinicians to properly interpret the health records (e.g., symptoms) as carrying the same meaning. Currently, several universal EHR providers, including Epic, Cerner, MEDITECH, and Allscripts, have joined to establish an interoperability initiative (http://www.beckershospitalreview.com/healthcare-information-technology/epic-cerner-meditech-dozens-more-make-interoperability-pledge-at-himss16-5-things-to-know.html). All of these vendors have agreed to overcome the challenges of medical record interchangeability, information sharing, and positive patient engagement. The settlement is a critical step towards constructing an allied healthcare system, where information is shared smoothly and in a secure manner across various EHR systems [115].

## 7. Discussion

In order to maximize preventive measures of serious but preventable diseases, it is critical to understand as much about the patient as early as possible. Generally, precautionary health interventions are simpler or more cost-effective than therapies implemented at a later stage. In addition, knowing patients’ individual characteristics is often helpful in providing effective and individualized therapy to a disease because individual patients can respond to the same treatment differently. Genomic medicine could change the path for preventing and treating human diseases. However, the translation of these advances into healthcare will rely critically on our ability to identify disease- and/or drug-associated clinically actionable variants and on our knowledge of the roles of the genetic alterations in the illness procedure.

To conduct pilot studies on incorporating genomic data into clinical care, a number of healthcare systems have developed bioinformatics infrastructures to process NGS data through a group of databases supplementary to the EHRs [116,117,118]. Most of the infrastructures are locally developed and proprietary, but this is because these centers are among the first healthcare providers to use genomic data in clinical care and there are no established infrastructures to meet their bioinformatics requirements. It requires substantial investment in resources and personnel for developing and deploying an efficient bioinformatics infrastructure for incorporating NGS data in clinical care. Thus, healthcare providers might want to consider cooperatively establishing a cloud computing service designed to store and process genomic data securely for the healthcare community. The cost of sequencing instruments may need to be taken into account as part of the infrastructure cost by clinical laboratories. Targeted sequencing instruments are less expensive and generate less data than the ones that perform WGS/WES. Therefore, more laboratories are likely to implement targeted sequencing before attempting to build a framework to support WGS/WES. For instance, a study conducted by Regeneron Genetics Center and the Geisinger Health System has highlighted the value of integrating genomic data and EHRs to uncover a genetic variant that results in reduced levels of triglycerides and a lower risk of coronary artery disease [119]. In addition, a study on the large-scale analysis of more than 50,000 exomes of patients and their EHRs by Regeneron and Geisinger has found clinically actionable genetic variants in 3.5% of individuals and several known and/or potential drug targets as well [120]. However, there are still challenges with integrating genomic data into EHRs in clinical practice, including reliable bioinformatics systems/pipelines that translate raw genomic data into meaningful and actionable variants, the role of human curation in the interpretation of genetic variants, and the requirement for consistent standards to genomic and clinical data [121].

A vital challenge of incorporating genomic data into clinical practice is the lack of standards for generating NGS data, bioinformatics processing, data storage, and clinical decision support. Standards could promote interoperability in data quality. Obedience to standards would enable the routine use of genomic data in clinical care. However, it is challenging to build standards when NGS technology and bioinformatics tools are frequently evolving. Furthermore, approaches to clinical decision support differ among healthcare institutions [116]. Appropriately integrating genomic data with EHRs for the discovery of clinically actionable variants can generate novel insights into disease mechanisms and provide better treatments. To improve our understanding on the nature of the disease from comprehensive EHRs, new methods such as ML, NLP, and other artificial intelligence approaches are needed. However, not all patients are likely to benefit from the use of Big Data in healthcare due to our current knowledge gaps on how to extract useful information from large-scale genomic and clinical data and how to interpret discovered variants properly. In the meantime, targeted therapies are not yet available for many important genes, and regulatory issues need to be solved before some useful bioinformatics tools can be applied to clinical setting.

## 8. Conclusions

In conclusion, as EHRs are exceptionally private, methods of protecting patient data need to make certain that patient information is only shared with those with authorized access. Even with the existing challenges, the prospective advantages that genomic data can bring to healthcare are much more important than the potential disadvantages. The increasing development of integrating genomic data with EHRs may cause concerns, but genomic data will certainly play an important role in advancing genomic medicine only if patient privacy and data security can be strictly protected.

## Figures and Tables

**Table 1:** Studies and efforts of leveraging genomic data and EHRs for genomic research/medicine.

Project	Start Year	Aims	Website	Country
deCODE genetics	1996	To utilize population-based genomic data and EHRs to investigate inherited causes of common diseases	http://www.decode.com/ [13]	USA
PMRP	2002	To enroll >20,000 participants to form a resource enabling researchers to study which genes cause diseases, which genes predict reactions to drugs, and how environment and genes work together to cause diseases	http://www.marshfieldresearch.org/chg/pmrp/ [14]	USA
I2B2	2004	To enable clinical researchers to use existing clinical data and genomic data for discovery research; to facilitate the design of targeted therapies for individual patients with diseases having genetic origins	http://www.i2b2.org/ [15]	USA
CKB	2004	To identify the complex interplay between genes and environmental factors on the risks of common chronic diseases	http://www.ckbiobank.org/ [16]	China
eMERGE	2007	To develop methods and best strategies for utilizing EHRs for genomic research in support of implementing genomic medicine	http://emerge-network.org/ [17]	USA
UK Biobank	2007	To improve the prevention, diagnosis, and treatment of a wide range of serious and life-threatening illnesses through a collection of 500,000 volunteers' biosamples and medical records	http://www.ukbiobank.ac.uk/ [18]	UK
GANI_MED	2009	To develop targeted strategies for the prevention, diagnosis, and therapy of diseases, tailored to the specific characteristics of an individual patient or a well-defined patient group. Specifically, these strategies should improve prediction models for health and disease outcomes and also avoid inefficient therapy strategies and adverse side effects	http://www2.medizin.uni-greifswald.de/gani_med/index.php?L=1&id=603 [19]	Germany
KP RPGEH	2009	To explore the genetic and environmental factors that influence common disease	http://www.rpgeh.kaiser.org/ [20]	USA
SCAN-B Initiative	2010	To improve survival and quality of life for breast cancer patients through the introduction of gene expression and genomic tumor profiling into the clinical routine for breast cancer	http://scan.bmc.lu.se/index.php/Main_Page [21]	Sweden
PGPop	2010	To understand how a person’s genetic make-up affects his or her response to medications	http://pgpop.mc.vanderbilt.edu/ [22]	USA
MVP	2011	To enroll one million volunteers and use their clinical and genetic data to improve health care for veterans	http://www.research.va.gov/mvp/ [23]	USA
Cancer 2015 Study	2015	To classify cancers molecularly using MPS to promote more targeted treatment of cancer patients and improve patient survival and outcomes	[24]	Australia
Precision Medicine Initiative	2016	To gain better insights into the biological, environmental, and behavioral influences for disease treatment and prevention that takes into account individual variability in genes, environment, and lifestyle by using the genomic and clinical data of a million Americans	https://www.nih.gov/precision-medicine-initiative-cohort-program [1]	USA

## Supplementary Materials

Supplementary materials can be found at www.mdpi.com/1422-0067/18/2/412/s1.
