# Supplementary material for: Big Data Analytics for Genomic Medicine
Source: Int J Mol Sci. 2017 Feb 15;18(2):412. doi: 10.3390/ijms18020412 (PMC5343946; doi:10.3390/ijms18020412)
Supplement: Supplementary file 1 [file ijms-18-00412-s001.pdf]

Table S1. Short-read sequence alignment programs.

Name	Description	Paired-end option	Use FASTQ quality	Gapped	Multi-threaded	License	Website
BarraCUDA	A GPGPU accelerated Burrows-Wheeler transform (FM-index) short read alignment program based on BWA, supports alignment of INDELs with gap openings and extensions.	Yes	No	Yes	Yes (POSIX Threads and CUDA)	GPL	http://seqbarracuda.sourceforge.net/
BBMap	Uses a short kmers to rapidly index genome; no size or scaffold count limit. Higher sensitivity and specificity than Burrows-Wheeler aligners, with similar or greater speed. Performs affine-transform-optimized global alignment, which is slower but more accurate than Smith-Waterman. Handles Illumina, 454, PacBio, Sanger, and Ion Torrent data. Splice-aware; capable of processing long INDELs and RNA-seq. Pure Java; runs on any platform. Used by the Joint Genome Institute.	Yes	Yes	Yes	Yes	BSD	http://sourceforge.net/projects/bbmap/
BFAST	Explicit time and accuracy tradeoff with prior accuracy estimation, supported by indexing the reference sequences. Optimally compresses indexes. Can handle billions of short reads. Can handle insertions, deletions, SNPs, and color errors (can map ABI SOLiD color space reads). Performs a full Smith Waterman alignment.				Yes (POSIX Threads)	GPL	http://bfast.sourceforge.net/
BigBWA	Tool to run the Burrows-Wheeler Aligner-BWA on a Hadoop cluster. It supports the algorithms BWA-MEM, BWA-ALN, and BWA-SW, working with paired and single reads. It implies an important reduction in the computational time when running in a Hadoop cluster, adding scalability and fault-tolerance.	Yes	Low quality bases trimming	Yes	Yes	GPL v3	https://github.com/citiususc/BigBWA
BLASTN	BLAST's nucleotide alignment program, slow and not accurate for short reads, and uses a sequence database (EST, sanger sequence) rather than a reference genome.						http://blast.ncbi.nlm.nih.gov/
BLAT	Made by Jim Kent. Can handle one mismatch in initial alignment step.				Yes (client/server).	Free for academic and non-commercial use.	http://www.soe.ucsc.edu/~kent/

Bowtie	Uses a Burrows-Wheeler transform to create a permanent, reusable index of the genome; 1.3 GB memory footprint for human genome. Aligns more than 25 million Illumina reads in 1 CPU hour. Supports Maq-like and SOAP-like alignment policies	Yes	Yes	No	Yes (POSIX Threads)	Artistic License	http://bowtie-bio.sourceforge.net/
HIVE-hexagon	Uses a hash table and bloom matrix to create and filter potential positions on the genome. For higher efficiency uses cross-similarity between short reads and avoids realigning non unique redundant sequences. It is faster than bowtie and BWA and allows INDELs and divergent sensitive alignments on viruses and bacteria as well as more conservative eukaryotic alignments.	Yes	Yes	Yes	Yes	Free for academic and non-commercial users registered to HIVE deployment instance.	https://hive.biochemistry.gwu.edu/dna.cgi?cmd=about
BWA	Uses a Burrows-Wheeler transform to create an index of the genome. It's a bit slower than bowtie but allows INDELs in alignment.	Yes	Low quality bases trimming	Yes	Yes	GPL	http://bio-bwa.sourceforge.net/
BWA-PSSM	A probabilistic short read aligner based on the use of position specific scoring matrices (PSSM). The aligner is adaptable in the sense that it can take into account the quality scores of the reads and models of data specific biases, such as those observed in Ancient DNA, PAR-CLIP data or genomes with biased nucleotide compositions.	Yes	Yes	Yes	Yes	GPL	http://bwa-pssm.binf.ku.dk/
CASHX	Quantify and manage large quantities of short-read sequence data. CASHX pipeline contains a set of tools that can be used together or as independent modules on their own. This algorithm is very accurate for perfect hits to a reference genome.				No	Free for academic and non-commercial use.	http://carringtonlab.org/resources/cashx
Cloudburst	Short-read mapping using Hadoop MapReduce				Yes (Hadoop MapReduce)	Artistic License	http://sourceforge.net/apps/mediawiki/cloudburst-bio/index.php
CUDA-EC	Short-read alignment error correction using GPUs.				Yes (GPU enabled)		http://www.nvidia.com/object/ec_on_tesla.html
CUSHAW	A CUDA compatible short read aligner to large genomes based on Burrows-Wheeler transform.	Yes	Yes	No	Yes (GPU enabled)	GPL	http://cushaw.sourceforge.net/
CUSHAW2	Gapped short-read and long-read alignment based on maximal exact match seeds. This	Yes	No	Yes	Yes	GPL	http://cushaw2.sourceforge.net/

	aligner supports both base-space (e.g. from Illumina, 454, Ion Torrent and PacBio sequencers) and ABI SOLiD color-space read alignments.						
CUSHAW2-GPU	GPU-accelerated CUSHAW2 short-read aligner.	Yes	No	Yes	Yes	GPL	http://cushaw2.sourceforge.net/
CUSHAW3	Sensitive and Accurate Base-Space and Color-Space Short-Read Alignment with Hybrid Seeding	Yes	No	Yes	Yes	GPL	http://cushaw3.sourceforge.net/
drFAST	Read mapping alignment software that implements cache obliviousness to minimize main/cache memory transfers like mrFAST and mrsFAST, however designed for the SOLiD sequencing platform (color space reads). It also returns all possible map locations for improved structural variation discovery.	Yes	Yes (for structural variation)	Yes	No	BSD	http://drfast.sourceforge.net/
ELAND	Implemented by Illumina. Includes ungapped alignment with a finite read length.						
ERNE	Extended Randomized Numerical alignEr for accurate alignment of NGS reads. It can map bisulfite-treated reads.	Yes	Low quality bases trimming	Yes	Multithreading and MPI-enabled	GPL v3	http://erne.sourceforge.net/
GASSST	Finds global alignments of short DNA sequences against large DNA banks				Multithreading	CeCILL version 2 License.	http://www.irisa.fr/symbiose/projects/gassst/
GEM	High-quality alignment engine (exhaustive mapping with substitutions and INDELs). More accurate and several times faster than BWA or Bowtie 1/2. Many standalone biological applications (mapper, split mapper, mappability, and other) provided.	Yes	Yes	Yes	Yes	Dual (free for non-commercial use); GEM source is currently unavailable	http://gemlibrary.sourceforge.net/
Genalice MAP	Ultra-fast and comprehensive NGS read aligner with high precision and small storage footprint.	Yes	Low quality bases trimming	Yes	Yes	Commercial	http://www.genalice.com/product/genalice-map/
Geneious Assembler	Fast, accurate overlap assembler with the ability to handle any combination of sequencing technology, read length, any pairing orientations, with any spacer size for the pairing, with or without a reference genome.				Yes	Commercial	http://www.geneiousserver.com/
GensearchNGS	Complete framework with user-friendly GUI to analyze NGS data. It integrates a proprietary high quality alignment algorithm as well as plug-	Yes	No	Yes	Yes	Commercial	http://www.phenosystems.com/www/i

	in capability to integrate various public aligners into a framework allowing to import short reads, align them, detect variants and generate reports. It is geared towards re-sequencing projects, namely in a diagnostic setting.						ndex.php/products/ gensearchngs
GMAP and GSNAP	Robust, fast short-read alignment. GMAP: longer reads, with multiple INDELs and splices (see entry above under Genomics analysis); GSNAP: shorter reads, with a single INDEL or up to two splices per read. Useful for digital gene expression, SNP and INDEL genotyping. Developed by Thomas Wu at Genentech. Used by the National Center for Genome Resources (NCGR) in Alpheus.	Yes	Yes	Yes	Yes	Free for academic and non-commercial use.	http://research-pub.gene.com/gmap/
GNUMAP	Accurately performs gapped alignment of sequence data obtained from next-generation sequencing machines (specifically that of Solexa/Illumina) back to a genome of any size. Includes adaptor trimming, SNP calling and Bisulfite sequence analysis.		Yes (also supports Illumina *_int.txt and *_prb.txt files with all 4 quality scores for each base)		Multithreading and MPI-enabled		http://dna.cs.byu.edu/gnumap/
iSAAC	iSAAC has been designed to take full advantage of all the computational power available on a single server node. As a result, iSAAC scales well over a broad range of hardware architectures, and alignment performance improves with hardware capabilities	Yes	Yes	Yes	Yes	BSD	https://github.com/sequencing/isaac_aligner
LAST	LAST uses adaptive seeds and copes more efficiently with repeat-rich sequences (e.g. genomes). For example: it can align reads to genomes without repeat-masking, without becoming overwhelmed by repetitive hits.	Yes	Yes	Yes	No	GPL	http://last.cbrc.jp/
MAQ	Ungapped alignment that takes into account quality scores for each base.					GPL	http://maq.sourceforge.net/
mrFAST and mrsFAST	Gapped (mrFAST) and ungapped (mrsFAST) alignment software that implements cache obliviousness to minimize main/cache memory transfers. They are designed for the Illumina sequencing platform and they can return all possible map locations for improved structural variation discovery.	Yes	Yes (for structural variation)	Yes	No	BSD	http://mrfast.sourceforge.net/

MOM	MOM or maximum oligonucleotide mapping is a query matching tool that captures a maximal length match within the short read.				Yes		http://mom.csbc.vcu.edu/
MOSAIK	Fast gapped aligner and reference-guided assembler. Aligns reads using a banded Smith-Waterman algorithm seeded by results from a kmer hashing scheme. Supports read ranging in size from very short to very long.				Yes		http://bioinformatics.bc.edu/marthlab/Mosaik
MPscan	Fast aligner based on a filtration strategy (no indexing, use q-grams and Backward Nondeterministic DAWG Matching)						http://www.atgc-montpellier.fr/mpscan/
Novoalign & NovoalignCS	Gapped alignment of single end and paired end Illumina GA I & II, ABI Color space & ION Torrent reads.. High sensitivity and specificity, using base qualities at all steps in the alignment. Includes adapter trimming, base quality calibration, Bi-Seq alignment, and option to report multiple alignments per read.	Yes	Yes	Yes	Multi-threading and MPI versions available with paid license.	Single threaded version free for academic and non-commercial use.	http://www.novocraft.com/
NextGENe	NextGENe® software has been developed specifically for use by biologists performing analysis of next generation sequencing data from Roche Genome Sequencer FLX, Illumina GA/HiSeq, Life Technologies Applied BioSystems' SOLiD™ System, PacBio and Ion Torrent platforms.	Yes	Yes	Yes	Yes	Commercial	http://softgenetics.com/NextGENe.html
NextGenMap	Flexible and fast read mapping program (twice as fast as BWA), achieves a mapping sensitivity comparable to Stampy. Internally uses a memory efficient index structure (hash table) to store the positions of all 13-mers present in the reference genome. Mapping regions where pairwise alignments are required are dynamically determined for each read. Uses fast SIMD instructions (SSE) to accelerate the alignment calculations on the CPU. If available, alignments are computed on the GPU (using OpenCL/CUDA) resulting in an additional runtime reduction of 20 - 50%.	Yes	No	Yes	Yes (POSIX Threads, OpenCL/CUDA, SSE)	Open Source	https://github.com/Cibiv/NextGenMap/wiki
Omixon	The Omixon Variant Toolkit includes highly sensitive and highly accurate tools for detecting SNPs and INDELs. It offers a solution to map	Yes	Yes	Yes	Yes	Commercial	http://www.omixon.com/

	NGS short reads with a moderate distance (up to 30% sequence divergence) from reference genomes. It poses no restrictions on the size of the reference, which, combined with its high sensitivity, makes the Variant Toolkit well-suited for targeted sequencing projects and diagnostics.						
PALMapper	PALMapper, efficiently computes both spliced and unspliced alignments at high accuracy. Relying on a ML strategy combined with a fast mapping based on a banded Smith-Waterman-like algorithm it aligns around 7 million reads per hour on a single CPU. It refines the originally proposed QPALMA approach.				Yes	GPL	http://www.fml.tuebingen.mpg.de/raetsch/suppl/palmapper
Partek	Partek® Flow software has been developed specifically for use by biologists and bioinformaticians. It supports un-gapped, gapped and splice-junction alignment from single and paired-end reads from Illumina, Life technologies Solid TM, Roche 454 and Ion Torrent raw data (with or without quality information). It integrates powerful quality control on FASTQ/Qual level and on aligned data. Additional functionality includes trimming and filtering of raw reads, SNP and INDEL detection, mRNA and microRNA quantification and fusion gene detection.	Yes	Yes	Yes	Multiprocessor/ Core, Client-Server installation possible	Commercial, FREE trial version	http://www.partek.com/
PASS	Indexes the genome, then extends seeds using pre-computed alignments of words. Works with base space as well as color space (SOLID) and can align genomic and spliced RNA-seq reads.	Yes	Yes	Yes	Yes	Free for academic and non-commercial use.	http://pass.cribi.unipd.it/
PerM	Indexes the genome with periodic seeds to quickly find alignments with full sensitivity up to four mismatches. It can map Illumina and SOLiD reads. Unlike most mapping programs, speed increases for longer read lengths.				Yes	GPL	https://code.google.com/p/perm/
PRIMEX	Indexes the genome with a kmer lookup table with full sensitivity up to an adjustable number of mismatches. It is best for mapping 15-60bp sequences to a genome.	No	No	Yes	No (multiple processes per search)		https://www.researchgate.net/publication/233734306_mex-0.99.tar

QPalma	Is able to take advantage of quality scores, intron lengths and computation splice site predictions to perform and performs an unbiased alignment. Can be trained to the specifics of a RNA-seq experiment and genome. Useful for splice site/intron discovery and for gene model building. (See PALMapper for a faster version).				Yes (client/server)	GPLv2	http://www.fml.tuebingen.mpg.de/raetsch/projects/qpalma
RazerS	No read length limit. Hamming or edit distance mapping with configurable error rates. Configurable and predictable sensitivity (runtime/sensitivity tradeoff). Supports paired-end read mapping.					LGPL	http://www.seqan.de/projects/razers.html
REAL, cREAL	REAL is an efficient, accurate, and sensitive tool for aligning short reads obtained from next-generation sequencing. The programme can handle an enormous amount of single-end reads generated by the next-generation Illumina/Solexa Genome Analyzer. cREAL is a simple extension of REAL for aligning short reads obtained from next-generation sequencing to a genome with circular structure.		Yes		Yes	GPL	http://sco.h-its.org/exelixis/real/
RMAP	Can map reads with or without error probability information (quality scores) and supports paired-end reads or bisulfite-treated read mapping. There are no limitations on read length or number of mismatches.	Yes	Yes	Yes		GPL v3	http://rulai.cshl.edu/rmap/
rNA	A randomized Numerical Aligner for Accurate alignment of NGS reads	Yes	Low quality bases trimming	Yes	Multithreading and MPI-enabled	GPL v3	http://iga-rna.sourceforge.net/
RTG Investigator	Extremely fast, tolerant to high INDEL and substitution counts. Includes full read alignment. Product includes comprehensive pipelines for variant detection and metagenomic analysis with any combination of Illumina, Complete Genomics and Roche 454 data.	Yes	Yes, for variant calling	Yes	Yes	Free for individual investigator use.	http://www.realtimegenomics.com/
Segemehl	Can handle insertions, deletions and mismatches. Uses enhanced suffix arrays.	Yes	No	Yes	Yes	Free for non-commercial use	http://www.bioinf.uni-leipzig.de/Software/segemehl/
SeqMap	Up to 5 mixed substitutions and insertions/deletions. Various tuning options and input/output formats.					Free for academic and non-	http://biogibbs.stanford.edu/~jiangh/SeqMap/

						commercial use.	
Shrec	Short read error correction with a suffix trie data structure.				Yes (Java)		http://www.informatik.uni-kiel.de/~jasc/Shrec/
SHRiMP	Indexes the reference genome as of version 2. Uses masks to generate possible keys. Can map ABI SOLiD color space reads.	Yes	Yes	Yes	Yes (OpenMP)	BSD derivative	http://compbio.cs.toronto.edu/shrimp
SLIDER	Slider is an application for the Illumina Sequence Analyzer output that uses the "probability" files instead of the sequence files as an input for alignment to a reference sequence or a set of reference sequences.						http://www.bcgsc.ca/platform/bioinfo/software/slider
SOAP, SOAP2, SOAP3 and SOAP3-dp	SOAP: Robust with a small (1-3) number of gaps and mismatches. Speed improvement over BLAT, uses a 12 letter hash table. SOAP2: using bidirectional BWT to build the index of reference, and it is much faster than the first version. SOAP3: GPU-accelerated version that could find all 4-mismatch alignments in tens of seconds per one million reads. SOAP3-dp, also GPU accelerated, supports arbitrary number of mismatches and gaps according to affine gap penalty scores.	Yes	No	SOAP3-dp:Yes	Yes (POSIX Threads), SOAP3, SOAP3-dp need GPU with CUDAsupport.	GPL	https://sourceforge.net/projects/soap3dp/
SOCS	For ABI SOLiD technologies. Significant increase in time to map reads with mismatches (or color errors). Uses an iterative version of the Rabin-Karp string search algorithm.				Yes	GPL	http://socs.biology.gatech.edu/
SparkBWA	SparkBWA is a tool that integrates the Burrows-Wheeler Aligner—BWA on an Apache framework running on the top of Hadoop. The current version of SparkBWA (v0.1, march 2016) supports the algorithms BWA-MEM and BWA-ALN. Both work with paired-end reads.	Yes	Low quality bases trimming	Yes	Yes	GPLv3	https://github.com/citiususc/SparkBWA
SSAHA and SSAHA2	Fast for a small number of variants.					Free for academic and non-commercial use.	http://www.sanger.ac.uk/Software/analysis/SSAHA2/
Stampy	For Illumina reads. High specificity, and sensitive for reads with INDELs, structural variants, or many SNPs. Slow, but speed	Yes	Yes	Yes	No	Free for academic and non-	http://www.well.ox.ac.uk/project-stampy

	increased dramatically by using BWA for first alignment pass).					commercial use	
SToRM	For Illumina or ABI SOLiD reads, with SAM native output. Highly sensitive for reads with many errors, INDELs (full from 0 to 15, extended support otherwise). Uses spaced seeds (single hit) and a very fast SSE/SSE2/AVX2/AVX-512 banded alignment filter. For fixed-length reads only, authors recommend SHRiMP2 otherwise.	No	Yes	Yes	Yes (OpenMP)	Open source	http://bioinfo.lifl.fr/yass/iedera_solid/storm/
Subread and Subjunc	Superfast and accurate read aligners. Subread can be used to map both gDNA-seq and RNA-seq reads. Subjunc detects exon-exon junctions and maps RNA-seq reads. They employ a novel mapping paradigm called "seed-and-vote".	Yes	Yes	Yes	Yes	GPL3	http://subread.sourceforge.net/ http://bioconductor.org/packages/release/bioc/html/Rsubread.html
Taipan	de-novo Assembler for Illumina reads					Free for academic and non-commercial use.	http://taipan.sourceforge.net/
UGENE	Visual interface both for Bowtie and BWA, as well as an embedded aligner	Yes	Yes	Yes	Yes	Open source, GPL	http://ugene.unipro.ru/
VelociMapper	FPGA-accelerated reference sequence alignment mapping tool from TimeLogic. Faster than Burrows-Wheeler transform-based algorithms like BWA and Bowtie. Supports up to 7 mismatches and/or INDELs with no performance penalty. Produces sensitive Smith-Waterman gapped alignments.	Yes	Yes	Yes	Yes	Commercial	http://www.timelogic.com/catalog/799/velocimapper
XpressAlign	FPGA based sliding window short read aligner which exploits the embarrassingly parallel property of short read alignment. Performance scales linearly with number of transistors on a chip (i.e. performance guaranteed to double with each iteration of Moore's Law without modification to algorithm). Low power consumption is useful for data center equipment. Predictable runtime. Better price/performance than software sliding window aligners on current hardware, but not better than software BWT-based aligners currently. Can cope with large					Free for academic and non-commercial use.	http://www.bcgsc.ca/platform/bioinfo/software/fpga-illumina-aligner

	numbers (>2) of mismatches. Will find all hit positions for all seeds. Single-FPGA experimental version, needs work to develop it into a multi-FPGA production version.					
ZOOM	100% sensitivity for a reads between 15 - 240bp with practical mismatches. Very fast. Support insertions and deletions. Works with Illumina & SOLiD instruments, not 454.			Yes (GUI) No (CLI).	Commercial	http://www.bioinfor.com/zoom/general/overview.html

Table S2. Variant annotation programs.

Tools	Description	External resources use	Website
SnpEff	SnpEff annotates variants based on their genomic locations and predicts coding effects. Uses an interval forest approach	ENSEMBL, UCSC and organism based e.g. FlyBase, WormBase and TAIR	http://snpeff.sourceforge.net/SnpEff_manual.htm
VEP	Provides the location of specific variants in individuals. Variants are calculated using sanger-style resequencing data	dbSNP, Ensembl, UCSC and NCBI	http://www.ensembl.org/
ANNOVAR	This tool is suitable for pinpointing a small subset of functionally important variants. Uses mutation prediction approach for annotation	UCSC, RefSe and Ensembl	http://www.openbioinformatics.org/annovar/
PhD-SNP	SVM-based method using sequence information retrieved by BLAST algorithm.	UniRef90	http://snps.biofold.org/phd-snp/
PolyPhen-2	Suitable for predicting damaging effects of missense mutations. Uses sequence conservation, structure to model position of amino acid substitution, and SWISS-PROT annotation	UniPort	http://genetics.bwh.harvard.edu/pph2/
MutationTaster	Suitable for predicting damaging effects of all intragenic mutations (DNA and protein level), including INDELs.	Ensembl, 1000 Genomes Project, ExAC, UniProt, ClinVar, phyloP, phastCons, nnsplice, polyadq (...)	http://www.mutationtaster.org/
SuSPect	An SVM-trained predictor of the damaging effects of missense mutations. Uses sequence conservation, structure and network (interactome) information to model phenotypic effect of amino acid substitution. Accepts VCF file	UniProt, PDB,Phyre2 for predicted structures, DOMINE and STRING for interactome	http://www.sbg.bio.ic.ac.uk/suspect/index.html
F-SNP	Computationally predicts functional SNPs for disease association studies.	PolyPhen, SIFT, SNPeffect, SNPs3D, LS-SNP, ESEfinder, RescueESE, ESRSearch, PESX, Ensembl, TFSearch, Consite, GoldenPath, Ensembl, KinasePhos, OGPET, Sulfinator, GoldenPath	http://compbio.cs.queensu.ca/F-SNP/
AnnTools	Design to Identify novel and SNP/SNV, INDEL and SV/CNV. AnnTools searches for overlaps with regulatory elements, disease/trait associated loci, known segmental duplications and artifact prone regions	dbSNP, UCSC, GATK refGene, GAD, published lists of common structural genomic variation, Database of Genomic Variants, lists of conserved TFBs, miRNA	http://anntools.sourceforge.net/
SNPit	Analyses the potential functional significance of SNPs derived from genome wide association studies	dbSNP, EntrezGene, UCSC Browser, HGMD, ECR Browser, Haplotter, SIFT	-
SCAN	Uses physical and functional based annotation to categorize according to their position relative to genes and according to linkage disequilibrium (LD) patterns and effects on expression levels	-	http://www.scandb.org/newinterface/about.html
SNAP	A neural network-based method for the prediction of the functional effects of non-synonymous SNPs	Ensembl, UCSC, Uniprot, UniProt, Pfam, DAS-CBS, MINT, BIND, KEGG, TreeFam	http://www.rostlab.org/services/SNAP
SNPs&GO	SVM-based method using sequence information, Gene Ontology annotation and when available protein structure.	UniRef90, GO, PANTHER, PDB	http://snps.biofold.org/snps-and-go/
LS-SNP	Maps nsSNPs onto protein sequences, functional pathways and comparative protein structure models	UniProtKB, Genome Browser, dbSNP, PD	http://www.salilab.org/LS-SNP

TREAT	TREAT is a tool for facile navigation and mining of the variants from both targeted resequencing and whole exome sequencing	-	http://ndc.mayo.edu/mayo/research/biostat/stand-alone-packages.cfm
SNPdat	Suitable for species non-specific or support non-model organism data. SNPdat does not require the creation of any local relational databases or pre-processing of any mandatory input files	-	https://code.google.com/p/snpdat/downloads/
NGS – SNP	Annotate SNPs comparing the reference amino acid and the non-reference amino acid to each orthologue	Ensembl, NCBI and UniProt	http://stothard.afns.ualberta.ca/downloads/NGS-SNP/
SVA	Predicted biological function to variants identified	NCBI RefSeq, Ensembl, variation databases, UCSC, HGNC, GO, KEGG, HapMap, 1000 Genomes Project and DG	http://www.svaproject.org/
VARIANT	VARIANT increases the information scope outside the coding regions by including all the available information on regulation, DNA structure, conservation, evolutionary pressures, etc. Regulatory variants constitute a recognized, but still unexplored, cause of pathologies	dbSNP,1000 genomes, disease-related variants from GWAS,OMIM, COSMIC	http://variant.bioinfo.cipf.es/
SIFT	SIFT is a program that predicts whether an amino acid substitution affects protein function. SIFT uses sequence homology to predict whether an amino acid substitution will affect protein function	PROT/TrEMBL, or NCBI's	http://blocks.fhcrc.org/sift/SIFT.html
FAST-SNP	A web server that allows users to efficiently identify and prioritize high-risk SNPs according to their phenotypic risks and putative functional effects	NCBI dbSNP, Ensembl, TFSearch, PolyPhen, ESEfinder, RescueESE, FAS-ESS, SwissProt, UCSC Golden Path, NCBI Blast and HapMap	http://fastsnp.ibms.sinica.edu.tw/
PANTHER	PANTHER relate protein sequence evolution to the evolution of specific protein functions and biological roles. The source of protein sequences used to build the protein family trees and used a computer-assisted manual curation step to better define the protein family clusters	STKE, KEGG, MetaCyc, FREX and Reactome	http://www.pantherdb.org/
Meta-SNP	SVM-based meta predictor including 4 different methods.	PhD-SNP, PANTHER, SIFT, SNAP	http://snps.biofold.org/meta-snp
